# Expanding Clinical Services to Hispanic/Latinx Dementia and Caregiver Populations in Central Virginia

**DOI:** 10.1002/alz.087571

**Published:** 2025-01-09

**Authors:** Antonio A Martinez Tlatenchi, Yenifer A Valera Sabino, M. Agustina Rossetti

**Affiliations:** ^1^ University of Virginia, Charlottesville, Virginia, VA USA; ^2^ University of Virginia, Charlottesville, VA USA

## Abstract

**Background:**

Hispanic/Latinx older adults have increased risk of developing Alzheimer’s disease, poor access to timely and quality dementia care, as well as limited access to caregiver support and interventions. We addressed these structural barriers at a local level in central Virginia in order to improve disparities in risk, early detection, and care.

**Method:**

Systematic expansion of services was undertaken by establishing a Spanish neuropsychological clinic, providing personalized scheduling services by providers to ensure appropriate follow‐up after referral is received, engaging in dementia specific community talks through a broader health system initiative (UVA Latinx Health Initiative), and facilitating dementia care coordination services for caregivers.

**Result:**

The expansion of services has resulted in increased care to the Spanish‐speaking population with minimal wait‐time from time of referral to neuropsychological services and assignment of a care coordinator. Additionally, community talks resulted in increased awareness of services in the local area.

**Conclusion:**

These efforts have addressed well documented barriers of the Latinx community including timely and adequate access to dementia specific care for patients and their caregivers.

